# Increased physical activity is not enough to recover astrocytic population from dark-rearing. Synergy with multisensory enrichment is required

**DOI:** 10.3389/fncel.2013.00170

**Published:** 2013-10-04

**Authors:** Harkaitz Bengoetxea, Naiara Ortuzar, Irantzu Rico-Barrio, José Vicente Lafuente, Enrike G. Argandoña

**Affiliations:** ^1^Laboratory of Experimental Neuroscience, Department of Neuroscience, Faculty of Medicine and Odontology, University of the Basque CountryLeioa, Spain; ^2^Unit of Anatomy, Department of Medicine, University of FribourgFribourg, Switzerland

**Keywords:** environmental enrichment, physical exercise, dark-rearing, astrocyte, cross-modal plasticity, neurogliovascular unit, angioglioneurins

## Abstract

Elimination of sensory inputs (deprivation) modifies the properties of the sensory cortex and serves as a model for studying plasticity during postnatal development. Many studies on the effects of deprivation have been performed in the visual cortex using dark-rearing as a visual deprivation model. It induces changes in all cellular and molecular components, including astrocytes, which play an important role in the development, maintenance, and plasticity of the cortex, mediated by cytokines which have been termed angioglioneurins. When one sense is deprived, a compensatory mechanism called cross-modal plasticity increases performance in the remaining senses. Environmental enrichment is so far the best-known method to compensate sensorial deprivation. The aim of this work is to study the effects of exercise alone, and of an enriched environment combined with exercise, on astroglial population in order to observe the effects of exercise by itself, or the potential synergistic effect during the rat visual system development. Pregnant Sprague-Dawley rats were raised in one of the following rearing conditions: in total darkness and enriched environment conditions with physical exercise, and in total darkness with voluntary physical exercise. Astrocytic density was estimated by immunohistochemistry for S-100β protein and quantifications were performed in layer IV. The somatosensorial cortex barrel field was also studied as control. Our main result shows that an enriched environment combined with voluntary physical exercise manages to reverse the negative effects induced by darkness over the astroglial population of both the visual and the somatosensory cortices. On the other hand, exercise alone only produces effects upon the astroglial population of the somatosensory cortex, and less so when combined with an enriched environment.

## INTRODUCTION

In most species sensory systems are immature at birth and their postnatal development is refined by sensory inputs which induce changes at behavioral, functional, cellular, and molecular levels (Greenough et al., 1987;Bengoetxea et al., 2012;Levelt and Hübener, 2012). Changes reach their maximum during the critical period (Hensch, 2004,^2005^), a time window specific for each sensorial cortex. For the visual system of rats, this period starts at the end of the third postnatal week, peaks during the fourth week, and ebbs away from the end of the sixth week (Fagiolini et al., 1994). Although most studies have been focused on the neuronal component of the cortex, experience-mediated changes occur in neurons, glia, and vessels that constitute the so called neurogliovascular unit (Argandoña et al., 2012). These three cellular elements are closely related, constituting *de facto* the functional unit of the CNS.

Maturation of the sensory system is not only mediated by neuronal activity and cortical circuits (Hensch and Fagiolini, 2005), but by a plethora of elements including angioglioneurins (Lafuente et al., 2012) – cytokines that have proliferative and protective effects on all components of the neurogliovascular unit. These molecules include factors initially described as neurotrophic factors, such as the brain derived neurotrophic factor (BDNF); angiogenic factors, such as the vascular endothelial growth factor (VEGF); or metabolic factors, such as the insulin-like growth factor (IGF) or erythropoietin (EPO;Zacchigna et al., 2008).

Modification of the properties in the sensory cortex by means of elimination of the sensory input (deprivation) is a widely used model for the study of plasticity during postnatal development (Fagiolini et al., 1994;Argandoña and Lafuente, 1996;Frasnelli et al., 2011). Most studies analyzing the effects of deprivation have been performed in the visual cortex using dark-rearing as a visual deprivation model. It has been described that dark-rearing causes changes in all neurogliovascular components, inducing a decrease in neuronal and astroglial population (Argandoña and Lafuente, 1996,^2000^;Argandoña et al., 2003), among others.

Despite the fact that glial cells are by far the most numerous brain cell types, their role has long been regarded as of passive support. Since the seventies it is well known that astrocytes are mainly responsible for the development and function of the blood–brain barrier (Peterson et al., 2011). Recently the “tripartite synapse” paradigm (Perea et al., 2009;Ben Achour and Pascual, 2012) demonstrates that astroctyes are structurally and functionally related to neurons in a bidirectional way. Thus, astrocytes play a crucial role in the development, maintenance, plasticity, and function of the cortex, taking part in the control of the balance between energy demand and vascular support in the so-called neuro-glio-vascular coupling (Figley and Stroman, 2011). Considering the pivotal role of astrocytes in brain homeostasis and the strong metabolic cooperation existing between neurons and astrocytes, astrocytic dysfunction might contribute to neurodegenerative processes or vice versa, being both cellular types important unto each other (McGann et al., 2012).

A common feature for all sensory deprivation is the compensatory cross-modal plasticity which leads to an increased performance in the remaining senses when someone is subjected to sensory deprivation (Rauschecker, 1995;Merabet and Pascual-Leone, 2010). When sensory deprivation occurs, the corresponding cortex can be reactivated by the sensory system. The best-known method for compensating visual deprivation is environmental enrichment. Rearing in complete darkness from birth has major effects on the development of the visual cortex which can be reverted if animals are dark-reared in complex environments (Bengoetxea et al., 2008).Bartoletti et al. (2004) showed that environmental enrichment promotes the consolidation of the visual cortical connections and the development of visual acuity in dark-reared rats, and compensates for the effects of dark-rearing on a chondroitin sulfate proteoglycan-based perineuronal net establishment of the visual cortex. Visual deprivation also has effects at a molecular level. Our previous work showed that levels of the archetypal angioglioneurin VEGF diminish with visual deprivation, while an enriched environment helps to increase these levels up to control values (Bengoetxea et al., 2008).

Exercise has been considered essential for enriched environments to induce structural and functional changes (Will et al., 2004;Xie et al., 2013), but its role is still somewhat controversial, as exercise has been demonstrated to have effects by itself (Mustroph et al., 2012). In contrast, some behavioral studies have described a lack of differences between inclusion and non-inclusion of exercise (Pietropaolo et al., 2006). Most of these studies have been performed at the hippocampus and few results have been reported on sensory cortical areas.

The goal of this work was an in-depth analysis of the cross-modal reorganization of the visual cortex of rats, during development, in order to determine if the recovery of electrical properties and the closure of the critical period described by other authors can be correlated to quantitative effects on the astrocyte population. Therefore, we aimed to isolate exercise from the enriched environment and compare the importance of exercise alone or the potential synergistic effect of a combination of an enriched environment with exercise on the recovery of the astroglial population in the visual and the somatosensory cortices.

## MATERIALS AND METHODS

### ANIMALS AND HOUSING

**FIGURE 1:**
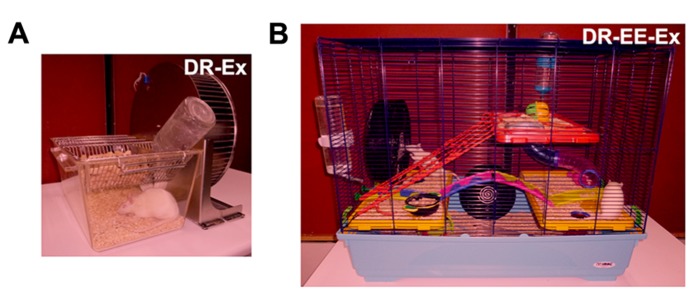
**Different dark-rearing conditions used.** **(A)** Dark-rearing with running wheel (exercise; DR-Ex); and **(B)** dark-rearing in an enriched environment and a running wheel (exercise; DR-EE-Ex). Litters were born in complete darkness and daily care tasks were performed under dim red light, as characterized in this figure.

Pregnant Sprague-Dawley rats were raised in different rearing conditions (**Figure 1**):

(1) Dark rearing with exercise (DR-Ex): rats raised in total darkness with unlimited access to a running wheel (2–3 rats per cage).
(2) Dark rearing in an enriched environment with exercise (DR-EE-Ex): rats raised in total darkness in enriched environment conditions and unlimited access to a running wheel. The enriched environment consists of a large cage (720 mm × 550 mm × 300 mm) furnished with colorful toys and differently shaped objects (shelters, tunnels) that were changed every 2 days (8–10 rats per cage; data for this group were retrieved from our previous work “Argandoña et al. (2009)” in order to reduce the use of animals).

During postnatal development, the following age groups were studied: P21, P28, P35, P42, P49, P56, and P63. Rats were raised from birth in each of the described rearing conditions. Weaning was carried out at P21, when males and females were separated and both sexes were used for experiments.

Every effort was made to minimize animal suffering and to reduce the number of animals used. All animal experiments were performed in accordance with European Community Council Directive (2010/63/EU), and approved by the Ethics Committee for Animal Welfare (CEBA) of the University of the Basque Country.

### FIXATION AND TISSUE PROCESSING

Rats were anesthetized with 6% chloral hydrate (performed under dim red light for DR groups), transcardially perfused with a fixative containing 4% paraformaldehyde in 0.1 M phosphate-buffered saline (PBS), and the brains were stored overnight at 4°C in fresh fixative. The following day, a thick block of occipital cortex containing the visual area, and a thick block of medial cortex containing the primary somatosensory area, were coronally removed with a Rodent Brain Matrix (Electron Microscopic Sciences, Hatfield, UK) and rinsed in 0.1 M PBS for 4 h. Samples for immunohistochemical analysis were embedded in paraffin, serially cut with a microtome into sections of 5 μm, and mounted on slides coated with 3-aminopropyltriethoxylane. On the other hand, samples assigned for volume analysis were stored in 30% sucrose in 0.1 M PBS, until the tissues sank, were cut using a cryostat microtome in 50 μm sections, and finally stored in free-floating chambers in 0.1 M PBS.

### IMMUNOHISTOCHEMISTRY

Paraffin sections were washed in 0.1 M PBS before inhibition of endogenous peroxidase activity using 4% H_2_O_2_ in 0.1 M PBS. Sections were washed in 0.1 M PBS prior to the incubation with blocking solution [5% bovine serum albumin (BSA) in 0.1 M PBS] for 1 h. Then, an anti-S-100β polyclonal antibody (Ref. S2644; working dilution 1:200; Sigma-Aldrich, USA) was added overnight at 4°C in 5% BSA in 0.1 M PBS containing 0.3% Triton X-100. Three washes in 0.1 M PBS followed, and a biotinylated anti-rabbit secondary antibody (Elite ABC kit, Vector Laboratories, USA) was added for 2 h at room temperature. Once rinsed, the sections were incubated with an avidin–biotin-peroxidase complex (Elite ABC kit, Vector Laboratories). A reaction product was detected using diaminobenzidine (0.25 mg/ml) as the chromogen and hydrogen peroxide solution (0.01%). Sections were processed with haematoxylin for the identification and localization of the primary visual cortex (V1) and the primary somatosensory cortex barrel field (S1BF). Sections were finally dehydrated and covered. As control for S-100β, we used brains of rats and humans that had been subjected to trauma. Negative controls in which the primary antiserum was omitted were also included in each staining run.

### NISSL STAINING

For Nissl staining, 50 μm-thick sections mounted on gelatine-coated slides were incubated in toluidine blue pH 4.1 for 1 min, dehydrated through a battery of alcohols with increased graduation, and coverslipped.****

### QUANTITATIVE ANALYSIS

To quantify any changes during development and to compare groups from both situations, a blind morphometric study was performed where the person who measured the sections did not know the particular characteristics of each case (neither the age of the rats nor whether they belonged to the experimental groups). To estimate the number of astrocytes per area, we counted the number of positive cell bodies present in an area delimited by a grid fixed in the eyepiece, excluding those intersected by the *X* and *Y* axes. The grid was a square with sides measuring 250 μm and the total surface area was therefore 62,500 μm^2^. The grid was randomly placed on the V1 between cortical layers III and V, ensuring that layer IV was included. Measurements were also taken from a non-visual area, e.g., S1BF on layer IV. The areas were selected with the aid ofPaxinos and Watson’s (1998) Rat Brain Atlas. A total of 56 animals, of both sexes, aged between P21 and P63 were used for DR-Ex group (*n *= 8 per age). Data for another 56 animals corresponding to the DR-EE-Ex group were retrieved from our previous work (Argandoña et al., 2009). Astrocytes were recognized by their morphological characteristics, i.e., S-100β-positive cell bodies with short positive cytoplasmic processes, and by their nuclear morphology. We did not count positive cells which did not fit the morphology of astrocytes. Measurements of each slice of the cortex were carried out on both the right and left hemispheres (eight fields were assessed on each hemisphere in each of the 10 slices taken per animal, i.e., 160 fields per animal) and the mean value per animal was calculated.

In order to rule out the possibility that changes in astroglial density might be due to changes in volume, the whole volume of layer IV was calculated for each region (V1 and S1BF), age, and condition using the Cavalieri method (Howard and Reed, 2005;Schmitz and Hof, 2005) included in the stereological module incorporated into the Mercator Software package for Computer Assisted Stereology (Explora Nova, La Rochelle, France).

**FIGURE 2:**
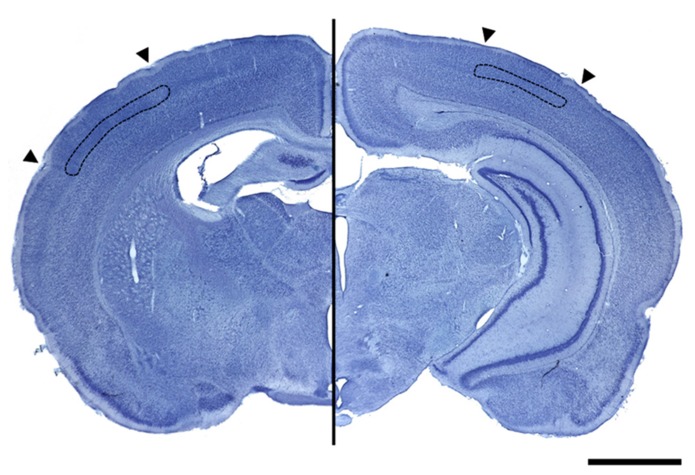
**Photographs of Nissl stained coronal sections containing the primary somatosensory cortex (left image) and the primary visual cortex (right image) delimited by arrowheads.** Images show, delimited in black, the layer IV that was used for volume calculation in both cortices (scale bar = 200 μm).

For this purpose, Nissl-stained sections were examined with a computer coupled to an Olympus BX41 microscope equipped with a motorized stage under the control of the Mercator software. Both V1 and S1BF, and the boundaries of layer IV, were delineated every 8th section usingPaxinos and Watson’s (1998) Rat Brain Atlas aided by the cytoarchitectonic characteristics of V1 and S1BF. The computer calculated the total volume by multiplying the area of the delineated ROIs by the serial section interval and the actual measured section thickness (**Figure 2**).

### STATISTICAL ANALYSIS

All statistical analyses were performed using SPSS statistical software (version 19.0 for IBM, Spain). Prior to analysis, data were examined for normal distribution using the Kolmogorov-Smirnov test and for homogeneity of variances using Levene’s test. The effects of the age period, experimental condition and their interaction were determined by two-way ANOVA with *posthoc* analysis (*posthoc* tests used the Bonferroni correction for equal variances or Tamhane’s T2 correction for unequal variances). In order to explore the effects of experimental conditions in greater depth, differences between them within age periods were evaluated using the Student’s *t*-test. Data are described as mean + SEM. Significance was declared at *p *< 0.05.

## RESULTS

**FIGURE 3:**
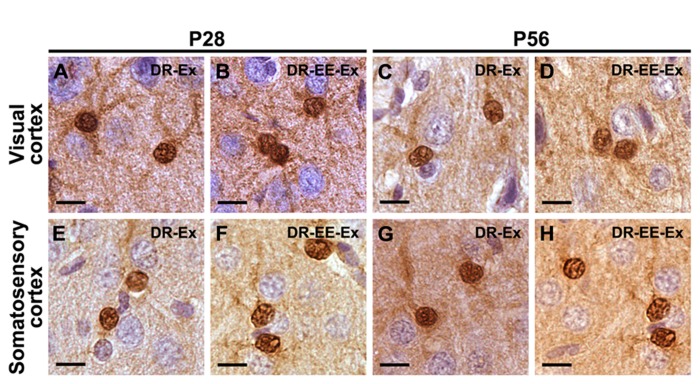
**S-100β staining shows that there are no morphological differences between astrocytes at any of the different ages and studied groups neither in the visual cortex nor in the somatosensory cortex.(A)** P28 DR-Ex visual cortex; **(B)** P28 DR-EE-Ex visual cortex; **(C)** P56 DR-Ex visual cortex; **(D)** P56 DR-EE-Ex visual cortex; **(E)** P28 DR-Ex somatosensory cortex; **(F)** P28 DR-EE-Ex somatosensory cortex; **(G)** P56 DR-Ex somatosensory cortex; **(H)** P56 DR-EE-Ex somatosensory cortex (scale bar = 10 μm).

**FIGURE 4:**
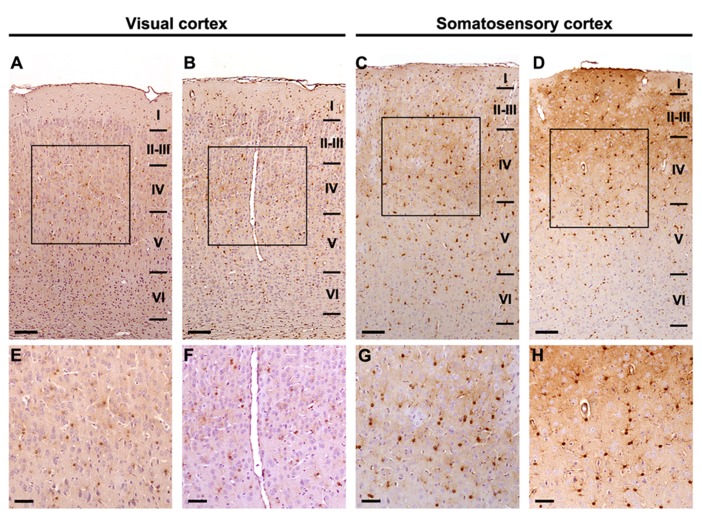
**Stereological analysis is required to discern astrocyte density differences between experimental conditions.** Images at different magnifications express the need for stereological quantification in order to elucidate numerical differences. S-100β positive astrocytes: **(A, E)** P28 DR-Ex visual cortex; **(B, F)** P28 DR-EE-Ex visual cortex; **(C, G)** P28 DR-Ex somatosensory cortex; **(D, H)** P28 DR-EE-Ex somatosensory cortex (scale bar = 100 μm in **A**, **B**, **C**, **D**; and 50 μm in **E**, **F**, **G**, **H**).

Immunoreactivity for S-100β was similar at all different ages both in the primary visual cortex and the primary somatosensory cortex, where strongly stained cell bodies and star-shaped processes were found across all cortical layers. No morphological differences between the studied groups were found at any of the different ages (**Figure 3**) and no quantitative difference could be guessed by microscopical analysis (**Figure 4**). Thus, we performed an unbiased stereological analysis in order to determine a numerical difference in astroglial population.

### ASTROGLIAL DENSITY

The main result is a statistically significant difference in astroglial density of the DR-EE-Ex group compared to the DR-Ex group, in both visual and somatosensory cortices, and across all the studied ages.

### PRIMARY VISUAL CORTEX

Two-way ANOVA revealed a significant interaction between age and experimental condition (*F *= 48.74, df = 6, *p = *0.000), as well as age (*F *= 26.88, df = 6, *p* = 0.000) and condition effects (*F *= 1248.85, df = 1, *p *= 0.000).

**FIGURE 5:**
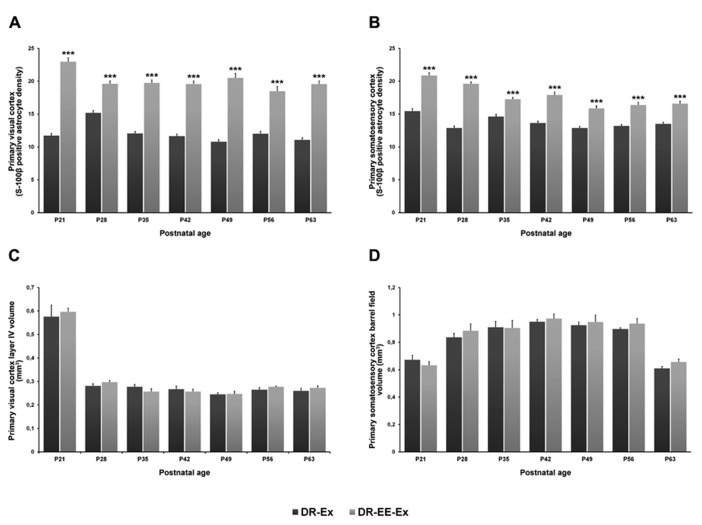
**Comparison of S-100β positive astrocyte density and cortical volume of the visual and somatosensory cortices between DR-Ex and DR-EE-Ex groups at each age considered.** **(A)** Primary visual cortex layer IV S-100β positive astrocyte density and **(B)** primary somatosensory cortex barrel field S-100β positive astrocyte density. The horizontal axis shows the age of the animals. The vertical axis shows astrocyte density per 62500 μm^2^ (mean ± SEM; ***p ≤ 0.001). **(C)** Primary visual cortex layer IV volume (mm^3^) and **(D)** primary somatosensory cortex barrel field volume (mm^3^). The horizontal axis shows the age of the animals. The vertical axis shows the cortical volume in mm^3^ (mean ± SEM).

Student’s *t*-test analysis was performed to study the differences between experimental conditions at each age period (**Figure 5**). Differences between DR-Ex and DR-EE-Ex groups were significant at all of ages analyzed (*p* = 0.000). The density of S-100β cells per area in the layer IV of the visual cortex was higher in the DR-EE-Ex group during the development with differences of 96% at P21 (11.70 ± 0.35 and 22.95 ± 0.59, DR-Ex vs. DR-EE-Ex), 30% at P28 (15.18 ± 0.37 and 19.60 ± 0.43), 64% at P35 (12.03 ± 0.33 and 19.72 ± 0.47), 68% at P42 (11.64 ± 0.28 and 19.56 ± 0.47), 90% at P49 (10.79 ± 0.33 and 20.51 ± 0.68), 54% at P56 (11.99 ± 0.38 and 18.46 ± 0.73) and 77% at P63 (11.06 ± 0.33 and 19.55 ± 0.49).

### PRIMARY SOMATOSENSORY CORTEX BARREL FIELD

Two-way ANOVA revealed a significant interaction between age and experimental condition (*F *= 11.05, df = 6, *p *= 0.000), as well as age (*F *= 28.06, df = 1, *p *= 0.000) and condition effects (*F *= 485.71, df = 1, *p *= 0.000).

Student’s *t*-test analysis between the two groups showed that, as in the visual cortex, differences were significant at all analyzed ages (*p* = 0.000), being DR-EE-Ex values higher than those for the DR-Ex group (**Figure 5**). Differences were 35% at P21 (15.44 ± 0.41 and 20.86 ± 0.41, DR-Ex vs. DR-EE-Ex), 52% at P28 (12.87 ± 0.32 and 19.60 ± 0.29), 18% at P35 (14.62 ± 0.34 and 17.26 ± 0.28), 31% at P42 (13.64 ± 0.28 and 17.90 ± 0.43), 23% at P49 (12.87 ± 0.25 and 15.87 ± 0.36), 24% at P56 (13.18 ± 0.23 and 16.38 ± 0.39) and 23% at P63 (13.49 ± 0.25 and 16.56 ± 0.41).

### CORTICAL VOLUME

Dark-rearing affected the volume of visual cortex layer IV, which was significantly larger in animals reared in standard laboratory conditions with a 12-h light/dark cycle than in all dark-reared groups (Argandoña et al., 2009). As we described in a previous work, no difference was found between DR-Ex and DR-EE-Ex groups. On the contrary, in the somatosensory cortex, the volume of layer IV increased in dark-reared groups (Argandoña et al., 2009), although no significant differences were found between them. Cortical volume of both DR-Ex and DR-EE-Ex groups was also similar.

### PRIMARY VISUAL CORTEX VOLUME

Two-way ANOVA revealed no differences or interactions between age and the experimental conditions (*F *= 1.59, df = 6, *p *= 0.164), as well as any condition effects (*F *= 0.86, df = 1, *p *= 0.357). However, age exerts significant changes over the primary visual cortex volume (*F *= 142.85, df = 6, *p *= 0.000).

There were no significant differences between the DR-Ex and DR-EE-Ex groups’ cortical volume at any of the studied ages (**Figure 5**). Values were 0.576 ± 0.049 and 0.596 ± 0.016 (DR-Ex vs. DR-EE-Ex, 4% difference, *p *= 0.872) at P21; 0.281 ± 0.009 and 0.297 ± 0.007 (6% difference, *p *= 1.000) at P28; 0.277 ± 0.010 and 0.257 ± 0.012 (-7% difference, *p *= 0.649) at P35; 0.267 ± 0.013 and 0.257 ± 0.010 (-4% difference, *p *= 1.000) at P42; 0.245 ± 0.007 and 0.247 ± 0.011 (1% difference, *p *= 1.000) at P49; 0.265 ± 0.009 and 0.277 ± 0.003 (5% difference, *p *= 1.000) at P56; and 0.260 ± 0.011 and 0.273 ± 0.008 (5% difference, *p *= 0.904) at P63.

### PRIMARY SOMATOSENSORY CORTEX BARREL FIELD

As occurred in the visual cortex, two-way ANOVA revealed no differences or interactions between age and the experimental conditions (*F *= 1.72, df = 6, *p *= 0.129), as well as any condition effects (*F *= 2.59, df = 1, *p *= 0.112). However, age exerts significant changes over the primary visual cortex volume (*F *= 25.70, df = 6, *p *= 0.000).

DR-Ex and DR-EE-Ex showed similar cortical values in the primary somatosensory cortex barrel field and statistically insignificant differences across all the ages studied (**Figure 5**). Values were 0.672 ± 0.032 and 0.632 ± 0.027 (DR-Ex vs. DR-EE-Ex, -6% difference, *p *= 1.000) at P21; 0.837 ± 0.028 and 0.884 ± 0.050 (6% difference, *p *= 0.648) at P28; 0.910 ± 0.042 and 0.905 ± 0.055 (-1% difference, *p *= 1.000) at P35; 0.950 ± 0.017 and 0.973 ± 0.033 (2% difference, *p *= 1.000) at P42; 0.925 ± 0.023 and 0.948 ± 0.050 (3% difference, *p *= 1.000) at P49; 0.897 ± 0.010 and 0.936 ± 0.038 (4% difference, *p *= 0.075) at P56; and 0.612 ± 0.013 and 0.2656 ± 0.022 (8% difference, *p *= 0.245) at P63.

## DISCUSSION

Visual deprivation induced by dark-rearing produces a down-regulation of the astroglial population of the visual cortex, related to a delay in the development of the neurogliovascular unit elements (Bengoetxea et al., 2012). Our study shows that this effect can be partially reverted by a compensatory mechanism stimulating other areas such as the somatosensory and the motor cortices but, for a full recovery, a combination of both these strategies; i.e., environmental enrichment (somatosensorial implementation) and exercise (motor implementation), is required. Indeed, neither our previous results with cognitive enrichment (Argandoña et al., 2009;Ortuzar et al., 2013) nor our current results with physical enrichment are able to reach a reversion as deep as the one we attained with the combination (Argandoña et al., 2009). These results stand in accordance with a previous study on the hippocampus describing additive effects on adult neurogenesis of both components of the environmental enrichment (Fabel et al., 2009). A recent study also reported that, although physical exercise is the major neurogenic and neurotrophic stimulus most likely mediated by the angioglioneurin BDNF, cognitive enrichment also has beneficial effects linked to an increased neuron survival (Bechara and Kelly, 2013). Recent results from our group showed that cognitive environmental enrichment recovers in part the effects of a focal injury, not only in the hippocampus, but also in the visual cortex (Ortuzar et al., 2010,^2011^, 2013). In addition, it has been recently demonstrated that cognitive enrichment also increases physical activity (Xie et al., 2013), thus providing synergistic effects on environmental enrichment, as it is almost impossible to dissociate all components.

Enriched environments have been deeply studied in recent past decades (Krech et al., 1960;Simpson and Kelly, 2011). Several studies have described how they induce effects from cellular (Sirevaag and Greenough, 1991;Leggio et al., 2005;Bengoetxea et al., 2008), molecular (During and Cao, 2006) or genetic (Burrows et al., 2011) levels, up to behavioral levels (Ortuzar et al., 2013), and it has been demonstrated that EE exhibits neuroprotective effects over many brain diseases (Lafuente et al., 2012) as well as restorative effects over sensory systems (Bartoletti et al., 2004). The enrichment strategy increases sensory, cognitive and motor stimulation and promotes neuronal activation, signaling and plasticity in all brain areas, such as the visual cortex or the somatosensory cortex (see review byPang and Hannan, 2013).

At present, there is a broad consensus about the compulsory role of increased physical activity in enrichment models (Kobilo et al., 2011). Exercise activates molecular and cellular cascades that support and maintain brain plasticity, like BDNF, and promotes angiogenesis, neurogenesis, and functional changes in the neuronal structure (Berchtold et al., 2010). These effects are not restricted to the motor cortex as it might have been anticipated. They are also reflected in the entire cortical layer as well as in the hippocampus, improving, in this case, cognitive skills (Nithianantharajah and Hannan, 2009;Kerr et al., 2010;Lista and Sorrentino, 2010). Comparing data obtained fromArgandoña et al. (2009) with our actual data, exercise counterbalances the deprivation effects produced by dark-rearing in the somatosensory cortex. Another point of interest is the difference between forced and voluntary exercise (Alomari et al., 2013). Previous studies have shown that forced exercise could be a source of stress which can mask the beneficial effects of exercise itself (Naylor et al., 2005;Griesbach et al., 2012). According to our observations, voluntary exercise is not limited to the use of the running wheel; there is also a high increase of physical activity when some elements such as platforms at different levels and tunnels connecting them are also included within the enriched environment.

Whereas most of the environmental enrichment paradigms include the use of a running wheel as part of the enrichment, this could mask results for the purpose of studying specifically the visual stimulation. In this study, exercise has been dissociated to analyze the effect over the cortical astrocyte population of physical activity alone, or combined with an enriched environment. In previous studies we have demonstrated the visual exclusiveness of an environmental enrichment lacking exercise, which was unable to compensate for the decrease in vascular density (Bengoetxea et al., 2008) and astrocyte density (Argandoña et al., 2009) in the visual cortex. Interestingly, comparing our previous data to data from this work, both exercise and enrichment combined increase the astrocyte density of the somatosensory cortex in rats raised in darkness, reversing the effects of dark rearing and obtaining values similar to those of the control group.

In the absence of visual stimulus, the exploration of novelty objects with the aid of whiskers reflects on the somatosensory cortex and amplifies the effects over other sensory cortices such as the visual one. Our results are consistent with a report that demonstrated physical exercise alone was less effective than its combination with an enriched environment (Fabel et al., 2009) although some authors have indicated that the inclusion of a running wheel as a part of the enriched environment does not alter the effects produced throughout the brain (Pietropaolo et al., 2006). Thus, according to our results, a complete environmental enrichment paradigm should include shelters, wheels, toys, tunnels, platforms at different levels and a metallic network to allow for climbing.

**FIGURE 6:**
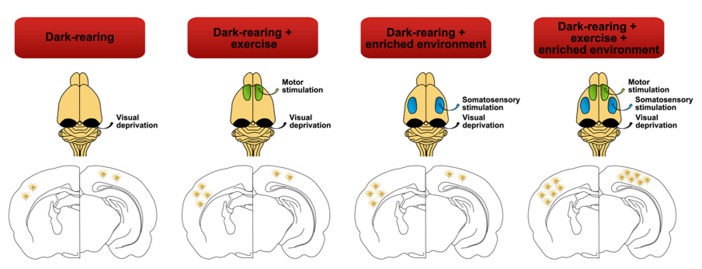
**Lack of visual stimulation (dark-rearing) not only exerts negative effects over the astrocyte density of the visual cortex but also affects other cortical areas such as the somatosensory cortex.** Strategies such as exercise or environmental enrichment, by themselves, may vary and recover the density of astrocytes in the somatosensory cortex almost to control group levels (Argandoña et al., 2009). But it is the combination of both paradigms that significantly increases astroglial cell number and therefore reverses the negative effects of dark rearing in both cortices.

The results of this work reinforce the data obtained previously (Argandoña et al., 2009). From the first stages of development, our results showed that the astroglial density in the DR-EE-Ex group, which combined both strategies, was statistically higher than in the control group. Neither exercise nor the enriched environment alone, reverted the negative effects induced by dark-rearing on the astroglial population, indicating that both strategies are complementary in producing cross-modal effects in the visual cortex (**Figure 6**). Moreover, we have observed that the increase in astroglial density is not attributable to an increase in volume. Dark-reared groups had a lower cortical volume than the control group, and the lack of difference between dark-reared groups means that any differences in astroglial density could not be attributable to variations in volume. Our results show a decrease in layer IV of the visual cortex volume in the control group at the beginning of the critical period which could be related to the decrease in the neuronal, vascular and astroglial densities (Argandoña and Lafuente, 2000;Argandoña et al., 2003,^2005^;Heck et al., 2008). Although there is an increase in astroglial density in the DR-EE-Ex group, it does not induce any changes in volume. Our results agree with previous findings showing a similar volume of layer IV in the visual cortex of young adult control rats (Yates et al., 2008).

In our behavioral paradigm, we should take into account that forced exercise may represent an additional source of stress along with visual deprivation, which may interfere with our results. In rats, some authors have described relevant changes in astroglial population when animals are exposed to different stress situations. For example,Sharma et al. (1992) have described that heat stress causes hypertrophy and reactivation of astrocytes. On other hand,Araya-Callís et al. (2012), have also described that chronic psychosocial stress downregulates GFAP expression in the hippocampus. Nevertheless, we can discard any effect of stress on astroglial population. According to our previous experience on dark-rearing (Argandoña and Lafuente, 1996,^2000^; ;Bengoetxea et al., 2008), as rats have been born in darkness and not introduced during development, no stress-induced behavioral changes have been detected.

Another point of interest is the observed difference in the somatosensory cortex. Visual deprivation encourages a greater use of the somatosensory skills, exerting effects on this cortex from P21 to P63. This increase is enhanced in environmentally enriched animals and the reinforcement is greater when the motor cortex is stimulated by the use of physical exercise. Unlike in the visual cortex, the volume of the somatosensory cortex of rats reared in darkness was greater than the control group ones, but no statistically significant difference was observed between them. The volume of the somatosensory cortex of dark-reared rats increased since the early stages of development due to the aforementioned compensation. As the critical period for the somatosensory cortex occurs earlier than the visual one (during the first week of life), the effects of compensatory skills through the use of whiskers are noticeable right from the beginning of the study. In a recent paper, in human blind subjects,Weaver et al. (2013) have suggested that a high level of cross-modal activity not merely prevents the decrease in astrocyte levels that is normally seen in dark-reared animals, but might even have an effect analogous to the effects of environmental enrichment, resulting in an increase in the astrocyte population local to the occipital cortex. They also have described neurochemical differences between blind and sighted subjects such as higher levels of GABA, lower levels of choline and higher levels of creatine. All these results support the idea that neurovascular coupling is also altered by early blindness. Astrocytes are not only a major component of the neurogliovascular unit, inducing and maintaining BBB properties and releasing angioglioneurins (Argandoña et al., 2012), they also play a major role in synaptic transmission. In the last 25 years, many studies have revealed the existence of bidirectional communication between neurons and astrocytes and proposed the term “tripartite synapse” to indicate the astrocyte as the third element of the synapse (Araque et al., 1999), therefore, any element that interfere with cortical function, such as visual deprivation, modifies all the elements of the neurogliovascular unit, also inducing changes in the tripartite synapse.

## CONCLUSION

Exposure to deprived inputs has serious consequences during the postnatal development of the sensory cortices, which can be reverted by reactivating cortical plasticity using physiological strategies such as enriched environment and voluntary physical exercise. There is a cross-modal plasticity that leads to a compensatory upregulation of the nondeprived senses that even rescues the cortical deficits of the deprived one. Our main result shows that somatosensorial and physical stimuli have effects all over the cortex and can rescue the deficits of the astroglial population of the visual cortex produced by the dark-rearing. Neither of these two strategies is enough by itself to offset the decrease on the astroglial population. However, this can be reverted by a combination of the two.

## Conflict of Interest Statement

The authors declare that the research was conducted in the absence of any commercial or financial relationships that could be construed as a potential conflict of interest.
